# Molecular mechanism of direct electron transfer in the robust cytochrome-functionalised graphene nanosystem†

**DOI:** 10.1039/d1ra02419a

**Published:** 2021-05-25

**Authors:** Margot Jacquet, Małgorzata Kiliszek, Silvio Osella, Miriam Izzo, Jarosław Sar, Ersan Harputlu, C. Gokhan Unlu, Bartosz Trzaskowski, Kasim Ocakoglu, Joanna Kargul

**Affiliations:** Solar Fuels Laboratory, Centre of New Technologies, University of Warsaw Banacha 2C 02-097 Warsaw Poland j.kargul@cent.uw.edu.pl; Chemical and Biological Systems Simulation Lab, Centre of New Technologies, University of Warsaw Banacha 2C 02-097 Warsaw Poland; Department of Engineering Fundamental Sciences, Faculty of Engineering, Tarsus University 33400 Tarsus Turkey; Department of Biomedical Engineering, Pamukkale University TR-20070 Denizli Turkey

## Abstract

Construction of green nanodevices characterised by excellent long-term performance remains high priority in biotechnology and medicine. Tight electronic coupling of proteins to electrodes is essential for efficient direct electron transfer (DET) across the bio-organic interface. Rational modulation of this coupling depends on in-depth understanding of the intricate properties of interfacial DET. Here, we dissect the molecular mechanism of DET in a hybrid nanodevice in which a model electroactive protein, cytochrome *c*_553_ (cyt *c*_553_), naturally interacting with photosystem I, was interfaced with single layer graphene (SLG) *via* the conductive self-assembled monolayer (SAM) formed by pyrene–nitrilotriacetic acid (pyr–NTA) molecules chelated to transition metal redox centers. We demonstrate that efficient DET occurs between graphene and cyt *c*_553_ whose kinetics and directionality depends on the metal incorporated into the bio-organic interface: Co enhances the cathodic current from SLG to haem, whereas Ni exerts the opposite effect. QM/MM simulations yield the mechanistic model of interfacial DET based on either tunnelling or hopping of electrons between graphene, pyr–NTA–M^2+^ SAM and cyt *c*_553_ depending on the metal in SAM. Considerably different electronic configurations were identified for the interfacial metal redox centers: a closed-shell system for Ni and a radical system for the Co with altered occupancy of HOMO/LUMO levels. The feasibility of fine-tuning the electronic properties of the bio-molecular SAM upon incorporation of various metal centers paves the way for the rational design of the optimal molecular interface between abiotic and biotic components of the viable green hybrid devices, *e.g.* solar cells, optoelectronic nanosystems and solar-to-fuel assemblies.

## Abstract 2

Molecular mechanism of DET between graphene and cytochrome *c* depends on the metal in the bio-organic interface: Co enhances the cathodic current *via* electron hopping from graphene to haem, whereas Ni exerts the opposite effect *via* tunnelling.

## Graphical abstract

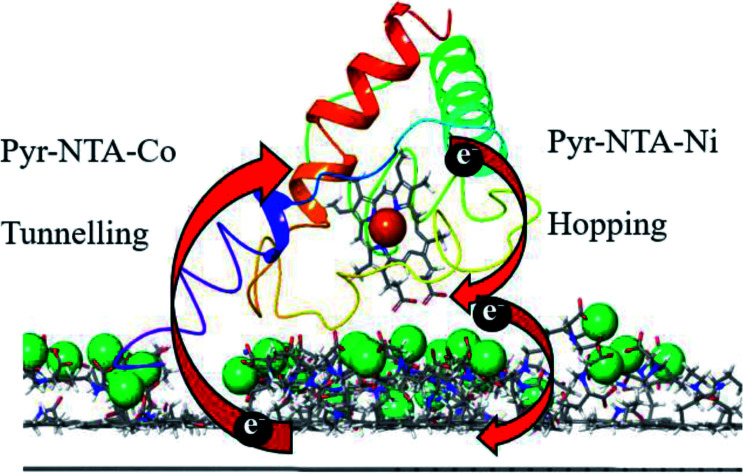

## Introduction

Construction of green nanodevices characterised by an excellent long-term performance (*e.g.*, high power output in case of solar-converting devices, or high selectivity and sensitivity towards the target molecules in the case of biosensors) remains a high priority in biotechnology and medicine. In the last decade the most studied materials which serve as a substrate for (bio)organic electrochemical systems are based on transition metal oxides (MoO_3_, V_2_O_5_, WO_3_),^1^ semiconductors (Al_2_O_3_, TiO_2_, Fe_2_O_3_)^2–4^ or silicon.^5,6^ Recently, the use of 2D materials, such as graphene and its derivatives plays an increasingly prominent role in competing with classic inorganic materials due to their high-performance physico-chemical properties, and relative easiness of synthesis.^7^ Graphene and its derivatives have been used as strong energy quenchers and electronic transducers in various fields, *e.g.*, optoelectronics, photovoltaics, photoelectrochemistry, and biomedicine,^8–12^ although the latter application remains to be elaborated due to the growing evidence of human health and environmental issues associated with this material.^13^ Nevertheless, such wide application of graphene stems from its large surface area, two-dimensionality, amenability to chemical and non-covalent functionalisation for binding of the sensing (bio)molecules, high electrical and heat conductivity, high transparency over the visible light spectral range, high mechanical strength and high biocompatibility.^8,14–16^

Recent works showed that non-covalent modification of graphene with pyrene (pyr) and its derivatives provides a promising route for immobilisation of a wide range of enzymes while retaining their activity.^17,18^ Notably, the pyr molecules forming the self-assembled monolayer (SAM) on graphene *via* π–π stacking, allowed for stabilisation and controlled oriented immobilisation of electroactive and catalytic light harvesting proteins, such as photosystem I (PSI), while maintaining high conductivity and structural integrity of the graphene monolayer.^19,20^ Moreover, the electronic properties of the pyr SAM itself can be tuned by the introduction of specific divalent transition metal redox centres, in order to improve the kinetics and directionality of direct electron transfer (DET) between graphene and pyr SAM.^20,21^

Cytochrome *c*_553_ (cyt *c*_553_) is a model electroactive protein involved in a fundamental process of natural oxygenic photosynthesis. The homologues of this protein are involved in mitochondrial respiration and apoptosis.^22^ In photosynthesis, this protein functions in the early events of solar light conversion, as it mediates ET between cytochrome b_6_f complex and the photo-oxidised P700^+^ chlorophylls forming the reaction centre of PSI.^23^ The X-ray analysis of the red algal cyt *c*_553_ protein, which is the robust electroactive protein used in this study, revealed that it is a Class I c-type cytochrome in which the redox-active prosthetic group is formed by haem covalently bound to Cys34 and Cys37 residues. The central Fe atom of the haem group displays octahedral coordination with His18 and Met58 axial ligands.^24^ The cyt *c*_553_ protein has been successfully applied in various types of photoactive nanodevices, in which the domain-specific molecular recognition between cyt *c*_553_ and PSI, that occurs *in vivo*, has been utilised for the specific orientation of PSI (reaction centre side toward the electrode surface) ensuring the preferred direction of ET through the system and formation of the oriented PSI photoactive monolayer on the electrode surface.^20^

Here, we report the comprehensive electrochemical and quantum mechanical characterisation of the DET processes in a cyt-functionalised single layer graphene (SLG) nanodevice in which the His_6_-tagged cyt *c*_553_ monolayer is used as the electro-responsive biotic component. The conductive interface between cyt *c*_553_ and the graphene monolayer is formed by an organic SAM composed of π–π-stacked pyr molecules functionalised with nitrilotriacetic acid (NTA) chelated to various transition metals (Co^2+^ or Ni^2+^). We demonstrate by three independent electrochemical approaches (cyclic voltammetry, photochronoamperometry and impedance spectroscopy) that efficient DET occurs between graphene and cyt *c*_553_ molecules and whose kinetics, directionality and stability depend on the metal redox centre incorporated into the bio-organic interface. The electrochemical data in conjunction with quantum mechanical simulations yielded the mechanistic models of DET occurring between graphene, pyr–NTA–M^2+^ SAM and cyt *c*_553_. Finally, we demonstrate the remarkable long-term stability of the cyt-based graphene nanodevice over the period of up to 5 months of interim illumination at ambient conditions.

## Experimental

### Materials characterisation and functionalisation of the graphene electrode with cytochrome *c*_553_

The FTO electrode was covered with SLG according to the procedure described previously.^20,25^ The quality and the number of graphene layers on FTO was examined using Raman scattering spectroscopy (WITec Alpha300 Raman microscope). The morphological structure of the SLG monolayer on the FTO surface and cross-sectional imaging of the FTO/SLG/pyr–NTA–M^2+^ electrodes was obtained by Field Emission Scanning Electron Microscope (FE-SEM, Zeiss Supra 55). Pyr–NTA moiety was synthesised as described in ref. 26 and used to modify the SLG layer by 1 h incubation of the FTO/SLG substrate in 2 mM solution of pyr–NTA in *N*,*N*-dimethylformamide (DMF). Subsequently, the electrode surface was thoroughly rinsed with DMF in order to remove unbound pyr–NTA molecules followed by 1 h incubation at room temperature (RT) with the 100 mM aqueous solution of NiSO_4_ or Co(NO_3_)_2_. The FTO/SLG/pyr–NTA–M^2+^ samples were analysed using X-ray Photoelectron Spectroscopy (Specs-Flex mode instrument) to check the formation of the organic SAM on the SLG surface. To optimise the detection, a first layer of Pt/Pd was deposited on the different samples. The elemental analysis of the FTO/SLG/pyr–NTA–M surfaces was carried out using energy-dispersive X-ray (EDX) spectroscopy module coupled to a Zeiss/Supra 55 FE-SEM. After M^2+^ ion ligation the electrode was functionalised with a 30 μM His_6_-tagged cyt *c*_553_ 19-AA peptide linker variant solution by a 2 h incubation at room temperature.^20,27^ Protein solutions were prepared in a 5 mM phosphate buffer with 25% glycerol (w/v), followed by a rinsing step with 5 mM phosphate buffer (pH 7.0) and air-dried. For cyclic voltammetry measurements of cyt present in the electrolyte buffer, the protein samples were concentrated to 61 μM in 5 mM phosphate buffer without glycerol using the VIVASPIN 6 concentrators (3000 MWCO, WITKO, Poland) with a speed of 2500 × *g* at 4 °C. The redox activity of the concentrated cyt was recorded with a Shimazu UV 1800 spectrophotometer *via* absorption difference spectroscopy analysis at room temperature, as described previously.^27^

### Electrochemical characterisation

A Versa STAT 3 (Princeton Applied Research, USA) electrochemical workstation connected with a halogen white light source (Schott, KL 2500 LCD, Germany) was used for electrochemical investigations. The EIS analysis was performed with a Metrohm Autolab B.V. potentiostat/galvanostat. All measurements were carried out in a Teflon-made three-electrode cell filled with 5 mM phosphate buffer (pH 7.0) as an electrolyte. The FTO/SLG substrate modified with a bioactive cyt *c*_553_ layer served as a working electrode (WE) with its geometric active surface area of 0.4185 cm^2^ connected through the copper tape with a conductive adhesive (6.4 mm width) in order to provide electrical contact. A silver/silver chloride electrode (Ag/AgCl, 3 M KCl) and glassy carbon rod (GC) were used as the reference (REF) and counter (CE) electrodes, respectively. The photochronoamperometry measurements were performed under aerobic conditions at −300 mV in the dark or under illumination of 100 mW cm^−2^ with a ‘light ON/OFF’ period of 30 s. The electrochemical impedance spectroscopy (EIS) analysis was performed at a frequency range of 0.01 Hz to 100 kHz using a 10 mA AC signal. For each electrode, two measurements with and without light were made. Cyclic voltammetry (CV) analysis was carried out in the dark in an argon-saturated electrolyte. From the cyclic voltammograms of FTO/SLG/pyr–NTA–M^2+^/cyt nanoassembly that were recorded at various scan rates, the surface coverage (*Γ*) of cyt *c*_553_ was estimated according to the following equation:1
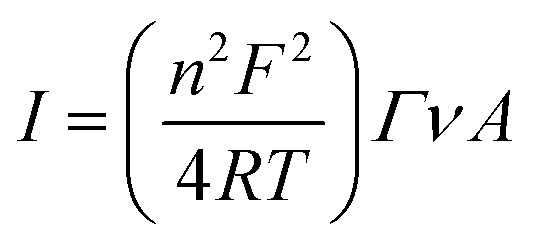
where *I* means the current peak of CV curves, *n* is the number of electrons transferred in the redox reaction (*n* = 1), *F* denotes the Faraday constant, *R* is the gas constant, *T* is the temperature (298 K), *ν* is the scan rate and *A* is the geometric active area estimated for the working electrode. All electrochemical results were recorded against an Ag/AgCl reference electrode in the absence of any exogenous artificial mediators.

The ET constant rate (*k*^0^_et_) values were extracted using the following equation:^28^2
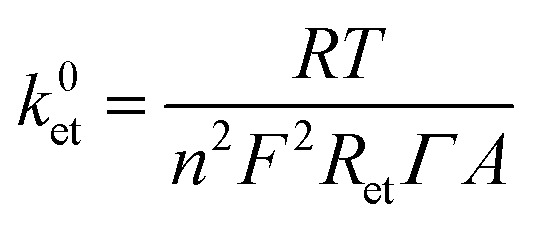
where *R* is the gas constant, *T* is temperature (298 K), *n* is number of electrons involved in the ET (*n* = 1), *F* is the Faraday constant, *R*_et_ denotes charge transfer resistance, *Γ* is the surface coverage and *A* is the geometric active area (0.4185 cm^2^).

### Computational methods

The model of the system considered in this study has been built considering a 6 × 6 nm^2^ SLG sheet on which 30 pyr–NTA molecules were physisorbed using a random pattern for the positioning of the pyrene moieties. To account for the anchoring of the linker to one of the pyr–NTA molecules, specific force-field parameters were considered (see ESI† for more details) for the coordination of the NTA–Ni^2+^/His_6_-tag moiety. The 19AA peptide linker was directly connected to the cyt holoprotein *via* its C-terminus as well as connected *via* the His_6_-tag to the NTA–Ni^2+^ molecule *via* two histidine residues (His2 and His3). At the starting point of the simulation, cyt was oriented with the haem group parallel to the SLG layer, at approximately 5 nm away from the SAM.

To assess the orientation and position of the cyt *c*_553_ on the SLG/SAM interface, classical MD simulations were carried out with the GROMACS 2018 program^29^ using the CHARMM36 force-field,^30^ with the time-step of 2 fs and the total simulation time of 300 ns at 300 K, in the NVT ensemble. Different geometrical analyses, carried out to assess the equilibration of the system, revealed the interface equilibration after 200 ns (see ESI†). Next, 56 snapshots have been extracted from the last 10 ns of MD and used for Quantum Mechanics/Molecular Mechanics (QM/MM) calculations (see ESI† for more details). Since the geometry of the assembly is only weakly affected by the nature of the metal centre considered, we replaced the metal cation (Ni^2+^ to Co^2+^) in the extracted frames for the QM/MM analysis, without performing additional MD simulations for the Co^2+^ system. Within the QM/MM method, the system was split into two parts: the haem/NTA pair was described at the DFT level of theory, while the cytochrome was described using the electrostatic embedding scheme, to assess the effect of an anisotropic environment (as the final device is in an all-solid state, water molecules and ions were not considered in this part of the computational study). The CAM-B3LYP functional^31^ and the LACV3P** basis set^32^ were used, as implemented in the Jaguar v.9.5 program.^33^ For each MD snapshot extracted, a single point calculation was performed, and electronic properties were analysed. The details of the computational methodology, which is similar to our previous work,^34^ are described in the ESI.†

## Results

### Preparation and structural analyses of the FTO/SLG devices

**Fig. 1:**
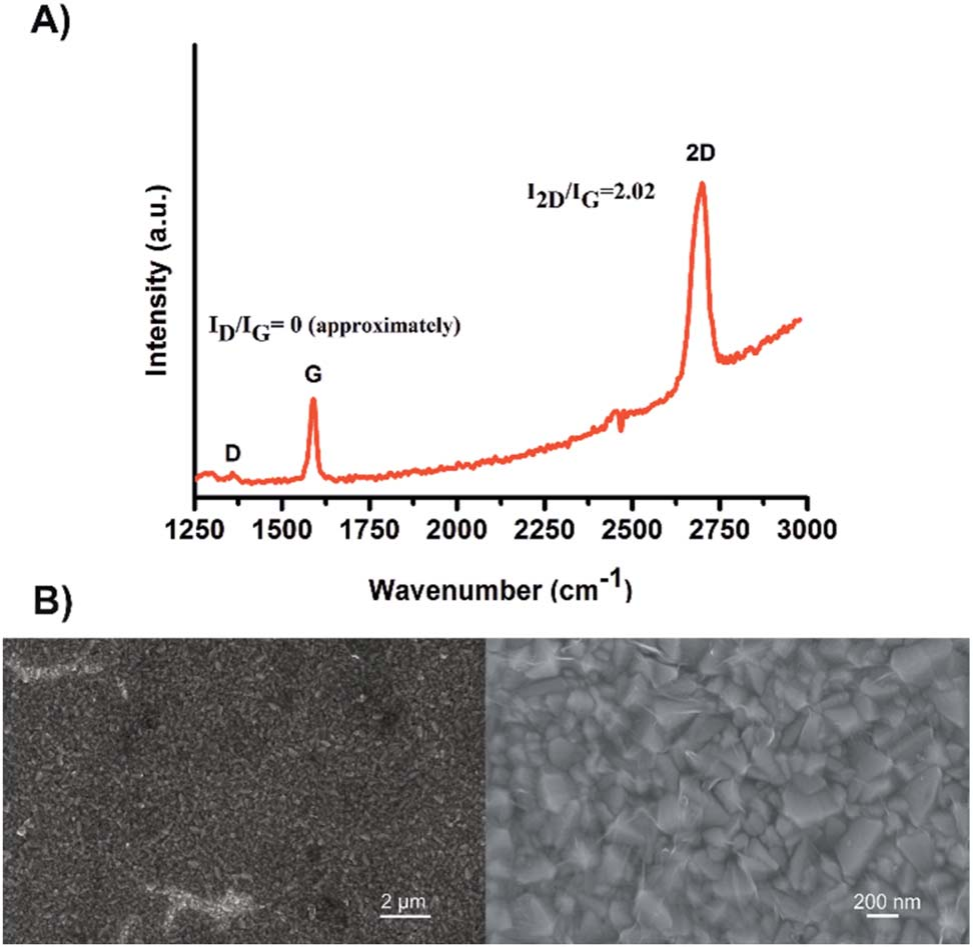
(A) A representative Raman spectrum of the SLG layer on FTO substrate. (B) SEM images of the FTO/SLG surface visualised at two different magnifications.

The different devices studied in this work were prepared according to the well-established procedures.^20^ The SLG layer was produced by chemical vapour deposition (CVD) on a highly pure cupper foil (99.999%) and then transferred onto the FTO substrate. The homogeneity and the quality of the SLG was confirmed by Raman spectroscopy and Scanning Electron Microscopy (SEM), as shown in Fig. 1. The obtained Raman spectrum (Fig. 1A) depicts the characteristic D, G and 2D signals of the SLG layer observed at 1357, 1589 and 2699 cm^−1^, respectively (*λ*_ex_ = 488 nm). The calculated *I*_D_/*I*_G_ peak ratio was approximatively 0.0025, reflecting a high quality of the defect-free graphene^35,36^ obtained in this study. Additionally, the well-symmetrical 2D signal and the 2D/G peak ratio of 2.02 confirm the intactness of the SLG deposited on the FTO surface. The morphological structure and homogeneity of the SLG were confirmed by SEM. Indeed, due to the absolute permeability of the SLG, the uniformity of the FTO material can be visualised (Fig. 1B).

**Fig. 2:**
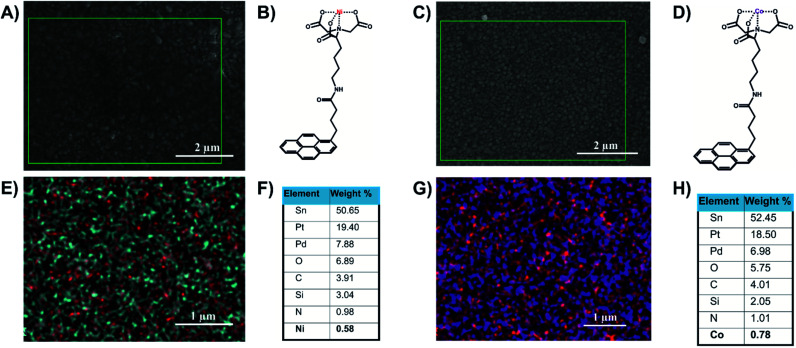
Structural and elemental analyses of the FTO/SLG/pyrene–NTA–M^2+^ surfaces. (A) A representative SEM image of FTO/SLG/pyr–NTA–Ni sample. (B) Chemical structure of the pyr–NTA–Ni molecule. (C) A representative SEM image of FTO/SLG/pyr–NTA–Co. (D) Chemical structure of the pyr–NTA–Co molecule. (E) EDX atomic mapping of the FTO/SLG/pyr–NTA–Ni surface, where the red and cyan spot represent Ni and N atoms, respectively. (F) Quantitative elemental analysis of the FTO/SLG/pyr–NTA–Ni surface derived from the EDX mapping. (G) EDX atomic mapping of FTO/SLG/pyr–NTA–Co surface, where the purple and red spots represent Co and N atoms, respectively. (H) Quantitative elemental analysis of FTO/SLG/pyr–NTA–Co surface derived from the representative EDX map. Pt and Pd signals originate from the conductive layer used for the EDX analysis.

The FTO/SLG surfaces were then functionalised with a pyrene–nitrilotriacetic acid self-assembled monolayer (pyr–NTA SAM) due to the π–π staking interactions between the sp^2^ lattice of the SLG and the pyrene moiety. The free NTA moiety was chelated with two different metals, cobalt and nickel, by immersing the surfaces in aqueous solutions of NiSO_4_ or Co(NO_3_)_2_. The obtained functionalised electrodes containing either cobalt (FTO/SLG/pyr–NTA–Co) or nickel (FTO/SLG/pyr–NTA–Ni) were characterised by X-ray Photoelectron Spectroscopy (Fig. S1, ESI†), SEM (Fig. 2A and C and S2, ESI†) and Energy-Dispersive X-ray spectroscopy (EDX) analyses (Fig. 2E and G).

The comparison of the XPS results between the bare FTO/SLG and the functionalised electrodes confirms the construction of the two organic interfaces (Fig. S1A†). The signals corresponding to Sn3d_5/2_, O1s, and C1s were found as expected in all the samples at 485.4 eV, 531.6 eV and 282.9 eV, respectively. For the functionalised surfaces, specific additional signals of the nitrogen from the pyr–NTA (Fig. S1E†) and from the different metals Ni2p_3/2_ (Fig. S1B†) and Co2p_3/2_ (Fig. S1C†) were found at 397.86 eV, 853.20 eV and 780.17 eV, respectively.

The EDX-SEM analyses (Fig. 2) allowed for the precise elemental mapping of the metalorganic interfaces, with the specific detection of nitrogen and nickel atoms for the FTO/SLG/pyr–NTA–Ni SAM (Fig. 2E) and the presence of nitrogen and cobalt atoms for the FTO/SLG/pyr–NTA–Co counterpart (Fig. 2G). Moreover, the obtained EDX maps and the corresponding quantitative elemental analyses (Fig. 2F and H) clearly confirm high homogeneity and high surface coverage of both types of samples.

Finally, the cyt *c*_553_ biocomponent was immobilised within the different devices following the binding of its C-terminal His_6_-tag to the metal–NTA SAM on the electrode surface.

### Electrochemical characterisation of the direct electron transfer in the hybrid graphene/cytochrome c nanoassemblies

Detailed kinetic characterisation of the intricate interfacial ET is a prerequisite for the rational design of (photo)electronic nanoassemblies in order to minimise charge recombination and short-circuiting processes that hamper the overall efficiency. In this study, we interfaced one of the main photosynthetic electroactive proteins, cyt *c*_553_ with single layer graphene (SLG) assembled on fluorine-doped tin oxide (FTO) substrate to obtain a model bioelectrochemical system for the detailed kinetic characterisation of DET (in the absence of external mediators) since this type of ET carries the high potential for the construction of viable green bioelectronic devices such as solar-to-fuel cells.

**Fig. 3:**
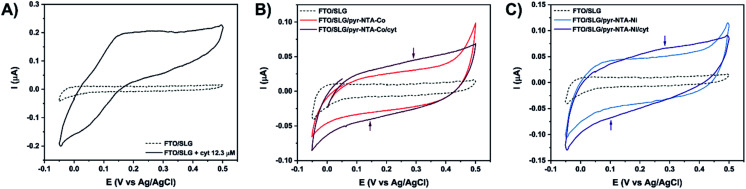
Cyclic voltammetry analysis of (A) bare FTO/SLG electrode (dashed line) in the presence of 12.3 μM cyt *c*_553_ (black). (B) Co-based hybrid nanoassemblies. (C) Ni-based hybrid nanoassemblies. Signals were recorded at 5 mV s^−1^ in Ar-saturated 5 mM phosphate buffer (pH 7.0) in the potential range from 0.5 V to −0.05 V *vs.* Ag/AgCl.

To ensure efficient DET between SLG and cyt *c*_553_ and to obtain a stable monolayer of this electroactive protein, we used a conductive organic SAM composed of pyr–NTA molecules. A similar strategy was used before to anchor cyt *c*_553_*via* its C-terminal His_6_-tag on various types of electrode materials.^25,27,37,38^ Previous studies demonstrated that incorporation of additional redox-active metal centres into the pyr–NTA SAM on graphene allows for manipulation of the output and directionality of the resultant photocurrents.^25^ In this study, we focused on the dissection of the precise molecular mechanism of DET within a much more complex molecular system comprising a graphene monolayer, pyr–NTA–M^2+^ SAM and the haem group of cyt *c*_553_ to determine the optimal supramolecular architecture of such nanoassembly for efficient DET. As a first step to assess the influence of each metal centre on DET, the redox behaviour of the cyt *c*_553_ protein, either suspended in the water-based electrolyte or captured on the SLG surface, was analysed by cyclic voltammetry (CV) using the FTO/SLG assembly as a working electrode (WE) (see Fig. 3).

Fig. 3A shows that the FTO/SLG electrode is redox inactive in the potential range from 0.5 V to −0.05 V *vs.* Ag/AgCl with only a small capacitive current observed. After the addition of cyt *c*_553_ in solution, a clear redox signature is observed between 0 V and 0.35 V, confirming the presence of DET between graphene and the haem group of cyt *c*. A broad oxidation peak is detected in the potential range of 0.15–0.33 V, while the reductive peak is more defined and observed around 0.02 V. We then proceeded to the electrochemical characterisation of the full FTO/SLG/pyr–NTA–M^2+^/cyt *c*_553_ bionanoassembly containing two distinct transition metal cations (Co^2+^ or Ni^2+^), since the previous study demonstrated the importance of these metallic redox centres for improvement of the kinetics and directionality of ET between graphene and the pyr-based SAM.^21^

Fig. 3B and C show cyclic voltammograms for the SLG/pyr–NTA–Co and SLG/pyr–NTA–Ni assemblies in the presence or absence of the cyt *c*_553_ thin layer. As expected, both types of samples show a higher capacitive current compared to the FTO/SLG sample due to the presence of the metallo-organic monolayer on the highly conductive SLG. In the applied potential range, the NTA–M^2+^ SAM is redox inactive (see blue and red traces in Fig. 3B and C). Binding of cyt *c*_553_ through the His_6_-tag, genetically introduced into the structure of this protein at the C-terminus, onto the pyr–NTA–M^2+^ SAM results in a subtle change of the redox behaviour between 0.05 V and 0.4 V. For the pyr–NTA–Co/cyt configuration (Fig. 3B), a slightly higher capacitive current is recorded, and the small redox peaks are present at 0.35 V and 0.14 V attributed to the Fe^III^/Fe^II^ couple in the haem group. Concerning the pyr–NTA–Ni/cyt assembly (Fig. 3C), the electrochemical signature of cyt is more visible with an oxidative peak at 0.28 V and a reductive peak at 0.1 V. Surprisingly, for both bio-functionalised electrodes, the redox peaks of cyt are positively shifted in comparison to those found for cyt *c*_553_ in the electrolyte solution. This observation could be explained by a stabilisation of the cyt electrochemical behaviour after its immobilisation on SLG *via* the pyr–NTA–M^2+^ SAM. Nevertheless, the electrochemical detection of the cyt redox peaks confirms tight electronic communication between the haem group and SLG *via* the pyr–NTA–M^2+^ metallo-organic interface.

In order to quantify the density of immobilised cyt *c*_553_, the surface coverage value (*Γ*) was calculated from the linear dependency (Fig. S3, ESI†) between the scan rate and current intensity of the cyt redox peaks obtained from the SLG/pyr–NTA–Ni/cyt and SLG/pyr–NTA–Co/cyt samples using eqn (1) (see Materials and methods). The surface coverage values for the cyt protein were estimated as 2.07 × 10^−11^ mol cm^−2^ and 2.08 × 10^−11^ mol cm^−2^, which corresponds to an ultrathin layer of this redox active protein. Notably, similar *Γ* values were reported for other types of (bio)organic and inorganic monolayers assembled on various types of electrode materials including graphene.^39–42^

**Fig. 4:**
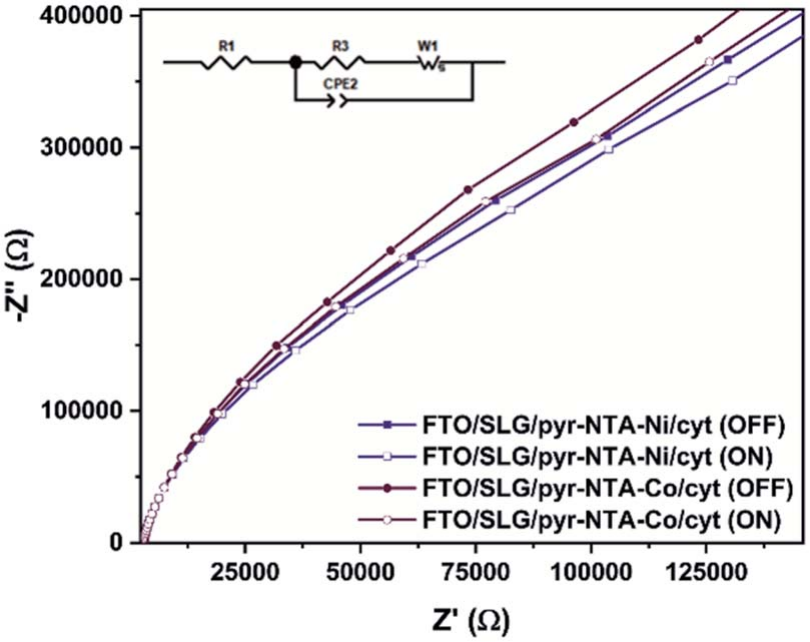
Nyquist plots in the dark and under illumination recorded for FTO/SLG/pyr–NTA–M^2+^/cyt *c*_553_ nanoassemblies. Inset: The equivalent circuit model used to fit the electrochemical impedance spectroscopy results.

To obtain a better insight into the ET process occurring between SLG, pyr–NTA SAM and cyt *c*_553_, electrochemical impedance spectroscopy (EIS) measurements were performed at the *E*_1/2_ recorded for immobilised cytochrome (Fig. 4). Table 1 presents fitted values of the EIS spectra to the equivalent circuit model (see Fig. 4, inset) represented by the electrolyte resistance (*R*_1_), the charge transfer resistance (*R*_2_) and the Warburg resistance associated with ions diffusion (*W*_s_) in parallel to the double layer capacitance expressed as the Constant Phase Element (CPE1) parameter (see Table S1 for additional parameters, ESI†). The comparison of Nyquist plots between both types of bio-electrodes shows a higher resistance for Co-based configuration regardless of the presence or absence of illumination. This observation is in accordance with the CV analysis (Fig. 3B and C), whereby the electrochemical detection of cyt was higher in the case of Ni-based SAM reflecting a smaller resistance from this type of interface. Upon illumination, both bio-functionalised electrodes reveal a decrease of their respective resistance due to the photoelectrochemical activity of both nanoassemblies.

**Table 1:** Resistance values obtained by EIS fitting based on the *R*_1_((*R*_2_*W*_1_)CPE1) model within different illumination conditions and calculated electron transfer kinetic constant *k*^0^_et_

Sample	Illumination	*R*_1_ (kΩ)	*R*_2_ (kΩ)	*k* ^0^_et_ (10^−2^ s^−1^)
Pyr–NTA–Ni/cyt	OFF	3.04	643	1.99
Pyr–NTA–Ni/cyt	ON	2.92	569	2.26
Pyr–NTA–Co/cyt	OFF	3.04	727	1.76
Pyr–NTA–Co/cyt	ON	2.91	642	1.99

The EIS and surface coverage data for the biofunctionalised electrodes were employed to calculate the *k*^0^_et_ kinetic constants of DET using the eqn (2) (see Materials and methods). The *k*^0^_et_ values (Table 1) are estimated to be 1.99 × 10^−2^ s^−1^ for SLG/pyr–NTA–Ni/cyt and 1.76 × 10^−2^ s^−1^ for SLG/pyr–NTA–Co/cyt systems under dark conditions and respectively 2.26 × 10^−2^ s^−1^ and 1.99 × 10^−2^ s^−1^ under illumination. These values are similar to the previously reported data.^43–45^ In line with the EIS results, the values of *k*^0^_et_ constant are smaller for the Co-based system. These observations are in accordance with quantum mechanical analyses, pointing on one hand towards a better capability of cathodic current generation in the case of Co redox centre, and on the other hand to anodic photocurrent generation in the presence of Ni-SAM.

**Fig. 5:**
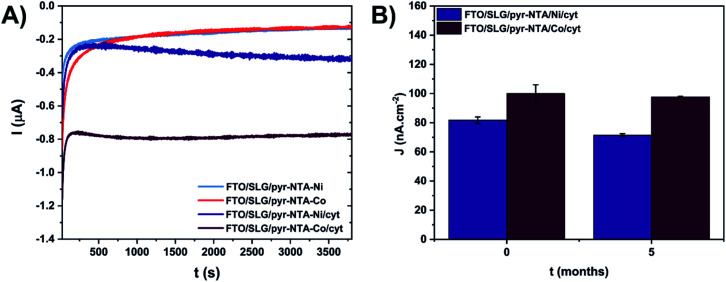
Short-term (A) and long-term (B) stability assessment of cytochrome c-based graphene nanoassemblies. (A) Current values recorded under continuous standard solar light illumination (1 sun) for 1 hour. (B) Current density values (*j*) recorded during 30 s. of ON/OFF illumination cycles (see Materials and methods). Photochronoamperometry was conducted for freshly prepared samples (*t* = 0) and after 5 months of storage at 4 °C in the dark. The measurements were performed at −300 mV *vs.* Ag/AgCl in 5 mM phosphate buffer (pH 7.0). Experiments for each system were carried out on 2 independent samples (*n* = 2).

The viability of the biohybrid devices depends to a large extent on their long-term stability. To this end, the short- and long-term stability of the cyt-based FTO/SLG/SAM assemblies was studied by photochronoamperometry, an approach that permits to record the photocurrent output (Fig. 5). The electrodes were initially subjected to continuous standard light illumination (1 sun) for up to 1 hour for the concomitant photocurrent measurement (short-term stability assessment, see Fig. 5A). The stability assessment revealed a similar electrochemical behaviour of the FTO/SLG/pyr–NTA–Ni and FTO/SLG/pyr–NTA–Co control electrodes, while the presence of cyt *c*_553_ on the FTO/SLG/pyr–NTA–Ni SAM results in a 2-fold higher photocurrent output compared to the control sample devoid of this protein (see Fig. 5A). On the other hand, the FTO/SLG/pyr–NTA–Co/cyt system produces the highest current value which is 5.5-times higher after 1 hour of constant illumination compared to the control devoid of cyt. In fact, the FTO/SLG/pyr–NTA–Co/cyt system showed the highest short-term stability compared to the other samples analysed in this study.

For the long-term stability assessment of the cyt-based nanoassemblies, the photocurrent output, produced within 30 s of ON/OFF illumination, was compared for the same samples after 5 months of storage under ambient conditions (Fig. 5B). For the freshly prepared biophotoelectrodes, the FTO/SLG/pyr–NTA–Co/cyt sample showed 22% higher current output (100 nA cm^−2^) compared to the FTO/SLG/pyr–NTA–Ni/cyt electrode (81.8 nA cm^−2^). After 5 months of storage, the respective current densities decreased to 97.7 nA cm^−2^ and 71.5 nA cm^−2^, which represents a mere 4.5% and 16.7% decrease of the photocurrent output for the Co- and Ni-containing biohybrid electrodes, respectively (Fig. 5B). These data clearly show a remarkable long-term stability of the Co-containing samples as well as the higher power output of the FTO/SLG/pyr–NTA–Co/cyt nanoassembly compared to the Ni-containing counterpart.

### Molecular mechanism of direct electron transfer in the cytochrome c-functionalised graphene nanoassemblies

In order to validate the obtained electrochemical data and obtain a better insight into the molecular mechanism of interfacial DET, we conducted Quantum Mechanical/Molecular Mechanics (QM/MM) simulations on the same nanosystems. The equilibration of the SLG/SAM/cyt interface from the molecular dynamics (MD) simulations shows that the cyt protein requires around 200 ns to fully relax over the SAM from the chosen starting orientation. Due to the presence of the 19 amino-acid long C-terminal peptide linker and its anchoring to one pyr–NTA–M^2+^ SAM molecule, the freedom of rotation and translation of the protein is strongly inhibited, leading to a limited number of thermodynamically allowed conformations. The average values of the gyration radius of 1.43 nm and the RMSD of 0.77 nm in the 200–300 ns time window suggest that the protein does not undergo significant conformational changes, thus most likely retaining its function (Fig. S2–S4, ESI†).

Within the 200–300 ns time frame, we extracted the minimum distance between the Fe^2+^ ion of the haem group and the Ni^2+^ cation of the SAM molecules, as well as the tilt angle between the haem group and the SLG, as they are the key parameters responsible for the DET efficiency of the whole conductive interface.^34^ The average minimum distance is 0.5 nm (Fig. S6, ESI†), while the tilt angle distribution peaks around 81 degrees, with a very narrow spread indicating the limited degrees of freedom for the movement of cyt c due to the presence of the anchoring peptide linker (Fig. S7, ESI†).

The extracted snapshots from the MD simulation are used to calculate the electronic properties of the pyr–NTA–M/haem interface. Among the 56 extracted snapshots, we observe two different sets; a major one composed of 48 frames with haem connected to one particular pyr–NTA–M molecule and a minor one of 8 frames with haem connected to a different pyr–NTA–M system (Fig. S8, ESI†). The difference between these distributions is the orientation of the pyr–NTA–M system with respect to the haem group. Importantly, the probability of occurrence of the two sets varies, with 86% of configurations belonging to the major set and only 14% representing the minor counterpart. Thus, we expect the first set to be more representative of the experimental nanodevice examined electrochemically in our study, although both should be considered to draw accurate conclusions.

**Fig. 6:**
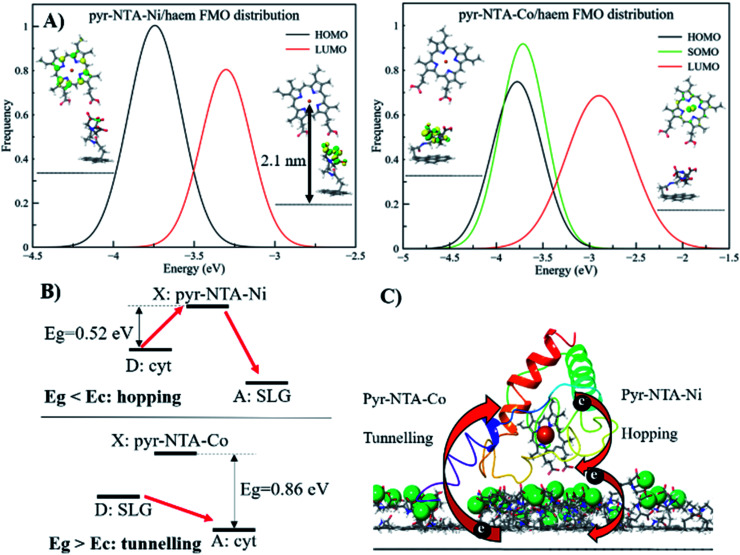
Mechanistic analysis of direct electron transfer in cytochrome c-based graphene nanoassemblies using QM/MM simulations. (A) Frontier molecular orbitals for the pyr–NTA–Ni/haem and pyr–NTA–Co/haem interfaces and their distribution. (B) Schematic representation of the different mechanisms underlying the DET for the SLG/pyr–NTA–Ni/cyt and SLG/pyr–NTA–Co/cyt interfaces. Red arrows indicate the different DET flow from the donor (D) to the X state and/or the acceptor (A) for the two pyr–NTA–M^2+^ interfaces. (C) Schematic visualisation of DET in the FTO/SLG/pyr–NTA–M^2+^/cyt *c* nanoassembly. Central Fe atom of haem (red ball) and Co and Ni redox centres (green balls) ligated to pyr–NTA SAM on SLG (side view of the graphene sp^2^ lattice in grey) are shown.

Due to the nature of the coordinating metal centres considered, two different electronic configurations were obtained: a closed-shell system for Ni and a radical system for the Co-containing nanoassembly. This, in turn, leads to very different frontier molecular orbitals (FMOs) localisation, as depicted in Fig. 6A and S9 (ESI).† From the pyr–NTA–Ni/haem FMO distribution, obtained from the different MD snapshots, we observe an average value of −3.74 ± 0.27 eV for the HOMO and −3.22 ± 0.17 eV for the LUMO, leading to an energy gap of 0.52 ± 0.26 eV (Fig. 6A). The rather high values for the standard deviations are due to the presence of thermal fluctuations arising from MD simulations. The analysis of the orbitals reveals that the HOMO is mainly localised over the haem group (with some occasional, small degrees of delocalisation over the NTA moiety), while the LUMO is always localised over the NTA–Ni moiety of the SAM molecule, thus explaining the small energy gap value obtained. This localisation of the FMOs indicates a DET from haem to pyr–NTA–Ni molecule, which should be enhanced by an external anodic bias. The same behaviour and DET direction have been found for the minor set (Fig. S9, details in ESI†). Our QM/MM findings are in the full agreement with the electrochemical results; in fact, when applying a cathodic bias, the generated current for this interface is lower, due to the counteraction of the external electric field and the internal DET.

A different scenario arises when the pyr–NTA–Co/haem interface is considered. Since it is a formally radical system, it is necessary to consider the presence of a semi-occupied molecular orbital (SOMO) in addition to HOMO and LUMO. From the FMO distribution, we observe that both HOMO and SOMO are very close in energy, with values of −3.83 ± 0.33 eV and −3.75 ± 0.29 eV, respectively, while the LUMO is found at −2.86 ± 0.33 eV, leading to an energy gap of 0.88 ± 0.34 eV (Fig. 6A). Interestingly, the presence of Co destabilises the LUMO with respect to the Ni system, while the HOMO is only slightly affected by the presence of the different cations, leading to an energy gap more than 1.5 times larger for Co than Ni, which might have dramatic consequences for the DET mechanism (see Discussion). For the second minor set of snapshots, the same trend has been found, although the difference in energy gap between the two interfaces is smaller, 3.43 and 3.11 eV for Co and Ni, respectively (see Fig. S9, ESI†). This is somehow unexpected, considering the different localisation of the FMOs when Co is present as the coordinating metal. In fact, the additional electron does not only affect the localisation of the orbitals but also the electron flow at the interface. In particular, for the pyr–NTA–Co/haem interface two different pictures, stemming from two different sets of FMOs, emerge: (i) both HOMO and SOMO are localised over the NTA–Co moiety of the SAM molecule while the LUMO is localised over the porphyrin fragment of the haem, and (ii) the occupied orbitals are localised over the porphyrin fragment and the LUMO is localised over the NTA–Co moiety. These two different configurations arise from the subtle changes in the conformation, orientation and distance of the two components of the interface due to thermal fluctuation from the MD simulation. Our calculations suggest a prevalence of the first case (up to 65%) in which the DET occurs from the pyr–NTA–Co to the haem, thus explaining the enhancement of generated current when an external cathodic bias is applied.

Interestingly, for the minor set of snapshots of the pyr–NTA–Co/haem interface only one distribution is obtained, in which the occupied orbitals are localised over the porphyrin fragment and the LUMO is localised over the pyr moiety of the SAM molecule (Fig. S9, ESI†), explaining the higher energy LUMO value obtained and, in turn, the bigger energy gap computed. As a result, for this complex interface we can consider the final DET flowing from the pyr–NTA–Co to the haem, in agreement with the experimental data. When a cathodic bias is applied, the external electric field and the DET flow along the same direction (as opposite to the case of the pyr–NTA–Ni/haem interface), strongly increasing the photogenerated current.

## Discussion

The long-range DET occurring in biomolecules can be described by either multistep hopping or by direct electron tunnelling mechanism. The determination of which mechanism dominate the DET is not a trivial task, especially for such a complex molecular system, and the key parameters to consider are the electronic properties of the redox centre. As the tunnelling rate decreases exponentially with the donor (D)–acceptor (A) distance, it becomes unfavourable at long distances. Yet, a high difference in redox potentials between D and the intermediate step X makes this state unlikely to be populated, suppressing the probability of the hopping mechanism. At the threshold value when the energy gap *E*_g_ (between the D and A, or between D and X in the D → X → A process) and the crossover barrier *E*_c_ are equal, there is an identical probability of direct tunnelling *versus* hopping, while hopping becomes predominant when *E*_g_ < *E*_c_ and *vice versa* (see Fig. S10, ESI†). To assess the DET mechanism, a few assumption are made: (i) the D →X, X → A and D → A steps have similar values of the reorganisation energy (this condition is commonly applied); (ii) moreover, *E*_c_ does not strongly depend on the reorganisation energy, and this term can be neglected (*λ* = 1 eV); (iii) the electronic coupling *V*_0_ values should be similar for different redox sites (in our case only one is present); (iv) the decay factor *β* ≈ 1 Å^−1^ in typical protein systems. This leads to the formulation of *E*_c_ as:
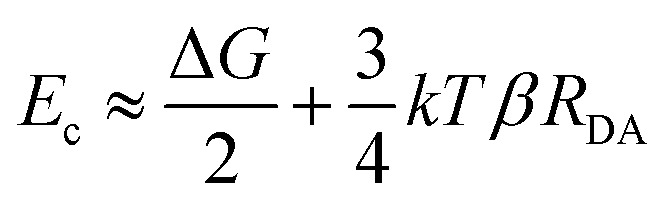
where *R*_DA_ is the donor–acceptor distance reported in angstrom, *E*_c_ and the driving force Δ*G* are in eV, *k* is the Boltzman constant and *T* = 300 K. The parameters to be considered are only the driving force and the D–A distance, together with the *E*_g_. The first term is easily obtained by CV experiments, while the other two are obtained by computational methods. Following the procedure reported in a previous work,^46^ we obtained values of Δ*G* = 0.2 V, *R*_DA_ = 24.8 Å and *E*_c_ = 0.58 eV for both interfaces containing Ni^2+^ and Co^2+^ cations. Our calculations yield *E*_g_ = 0.52 eV for pyr–NTA–Ni/haem and *E*_g_ = 0.88 eV for pyr–NTA–Co/haem interfaces, suggesting different DET mechanisms depending on the metal centre used to coordinate the pyr–NTA molecules (Fig. 6B). For the interface with Ni *E*_g_ < *E*_c_, therefore the hopping mechanism is dominating, while for the Co interface *E*_g_ > *E*_c_ and the tunnelling mechanism is the prevailing one (Fig. 6C). This semi-quantitative analysis is in agreement with the measured *k*^0^_et_ values of 2.26 × 10^−2^ s^−1^ for SLG/pyr–NTA–Ni/cyt and 1.99 × 10^−2^ s^−1^ for SLG/pyr–NTA–Co/cyt assemblies, indicating a faster DET for the Ni-containing bio-organic interface *via* a hopping mechanism and a slower tunnelling DET for the Co-containing interface.

## Conclusions

Our study provides electrochemical evidence for the occurrence of efficient DET between FTO/SLG substrate, organic interface composed of pyr–NTA SAM and the model electroactive protein of cyt *c*_553_. The electrochemical analyses (CV and EIS) in conjunction with QM/MM calculations demonstrate that DET between electrode, bio-organic interface and cyt *c* is facilitated when the Co redox centre is incorporated into the pyr-based SAM. The QM/MM calculations provide a firm mechanistic model for DET in both types of hybrid cyt/SLG-based nanoassemblies. We propose that two different DET mechanisms occur when the two different metal centres are considered. On one hand, when Ni^2+^ is the coordinating metal for binding of cyt, a net DET occurs from haem to SAM *via* a hopping mechanism, thus the application of an external cathodic potential is counteracted by the inner internal electron flow. On the other hand, the presence of Co^2+^ in the organic interface leads to the opposite scenario, where DET predominantly (more than 65% according to the QM/MM simulations) occurs from pyr–NTA SAM to the haem group and only the remaining pool of electrons flows in the opposite direction. Thus, the application of an external negative bias enhances the overall cathodic current generation from the SAM to the haem moiety *via* a tunnelling mechanism, as confirmed by lower experimental *k*^0^_et_ values obtained for this type of interface. The presence of a non-negligible amount of inverse charge flow from haem to pyr–NTA SAM (less than 14% according to the QM simulations) results in an increased current output.

In summary, our data demonstrate the successful application of Co as the redox metal centre in the organic interface based on pyr–NTA SAM for enhancement of the cathodic current from SLG to the haem group of cyt *c*. Importantly, the bionanoassemblies described here are characterised by the excellent long-term stability, with only a minor decrease of the power output over a period of up to 5 months of interim standard illumination at ambient conditions. The QM/MM-driven identification of the two distinct molecular mechanisms of DET occurring in the bio-organometallic interface with two different metallic centres paves the way for the rational design of the optimal molecular interface between abiotic and biotic components of the high-performance green hybrid devices ranging from solar cells, optoelectronic nanosystems and solar-to-fuel electrochemical cells.

## Author contributions

The manuscript was written through contributions of all authors. All authors have given approval to the final version of the manuscript.

## Conflicts of interest

There are no conflicts to declare.

## Supplementary Material

RA-011-D1RA02419A-s001
