# Supplementary material for: Molecular mechanism of direct electron transfer in the robust cytochrome-functionalised graphene nanosystem
Source: RSC Adv. 2021 May 25;11(31):18860–9. doi: 10.1039/d1ra02419a (PMC9033600; doi:10.1039/d1ra02419a)
Supplement: RA-011-D1RA02419A-s001 [file RA-011-D1RA02419A-s001.pdf]

Electronic Supplementary Material (ESI) for RSC Advances.
This journal is © The Royal Society of Chemistry 2021

Supplementary Information

Molecular mechanism of direct electron transfer in the robust cytochrome-functionalised graphene nanosystem

Margot Jacquet[a]†, Małgorzata Kiliszek[a]†, Silvio Osella[b]†, Miriam Izzo[a], Jarosław Sar[a], Ersan Harputlu[c], C. Gokhan Unlu[d], Bartosz Trzaskowski[b], Kasim Ocakoglu[c], and Joanna Kargul[a]*

[a] *Solar Fuels Laboratory, Centre of New Technologies, University of Warsaw, Banacha 2C, 02-097 Warsaw, Poland.*
[b]*Chemical and Biological Systems Simulation Lab, Centre of New Technologies, University of Warsaw, Banacha 2C, 02-097 Warsaw, Poland.*
[c]*Department of Engineering Fundamental Sciences, Faculty of Engineering, Tarsus University, 33400, Tarsus, Turkey.*
[d]*Department of Biomedical Engineering, Pamukkale University, TR-20070 Denizli, Turkey.*

† these authors contributed equally.
* corresponding author: Joanna Kargul, E-mail: j.kargul@cent.uw.edu.pl

Table of content

1. XPS and SEM analyses of the FTO/SLG/pyr-NTA-M^{2+} surfaces……………………………..……………….……………….2
2. Additional electrochemical data…………………………..……………………………………………………………………4
3. Computational details…….4

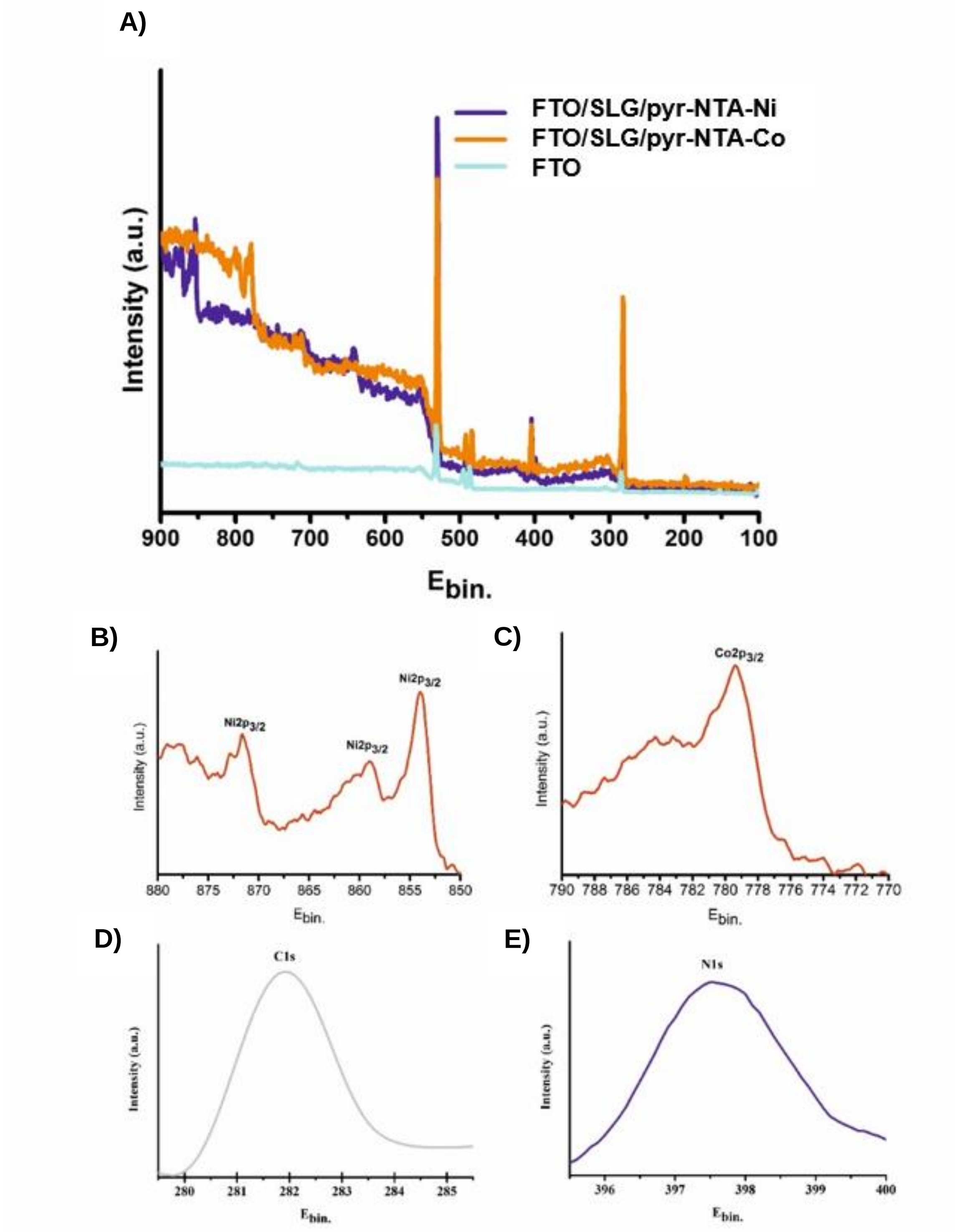

Fig. S1. XPS analysis of FTO/SLG/pyr-NTA-M^{2+} electrode surfaces. A) Broad-scan XPS spectra of FTO/SLG/pyr-NTA-M^{2+} and bare FTO surfaces. B) Ni2p3/2 XPS spectra of FTO/SLG/pyr-NTA-Ni. C) Co2p3/2 XPS spectra of FTO/SLG/pyr-NTA-Co. D) and E) C1s, N1s XPS spectra obtained from both Ni- and Co-based electrodes.

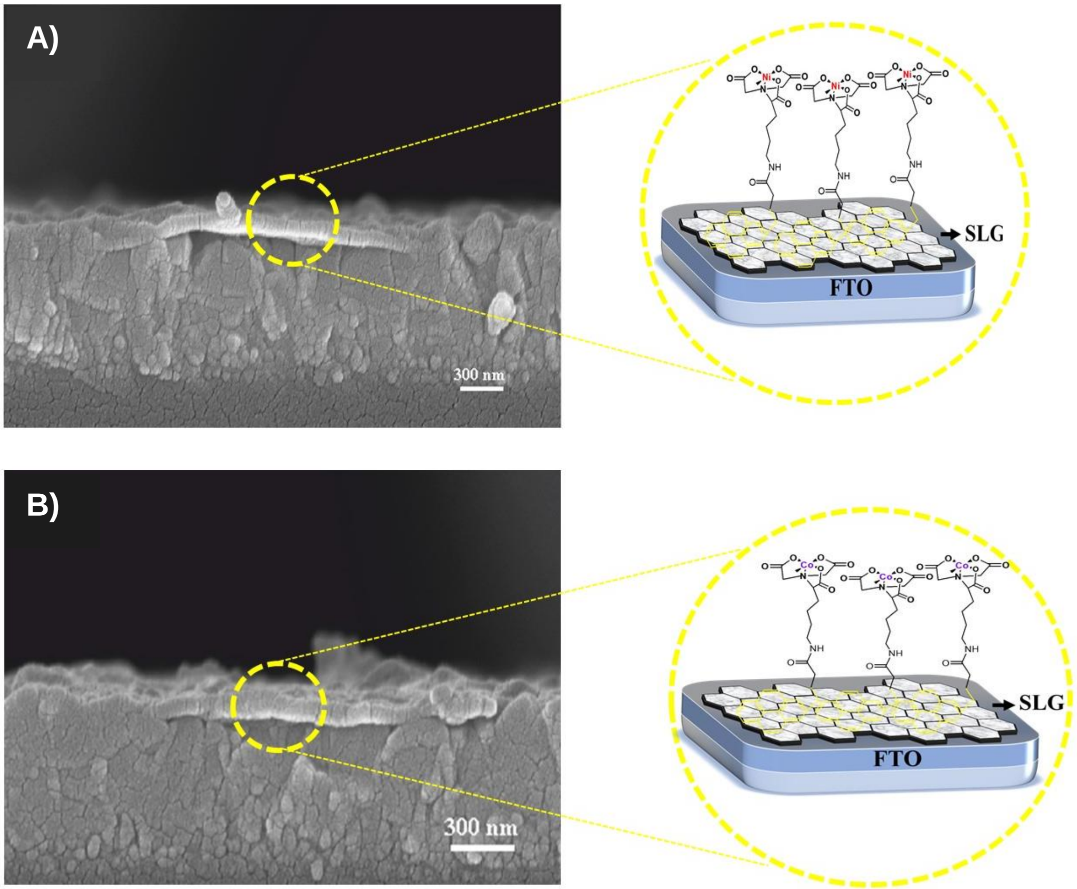

Fig. S2. Cross-sectional SEM imaging of the FTO/SLG/pyr-NTA-M^{2+} electrodes. A) Nickel-based electrode. B) Cobalt-based electrode. Insets: 3D organisation of the ultrathin pyr-NTA-M^{2+} SAM at an atomic scale.

Additional electrochemical data

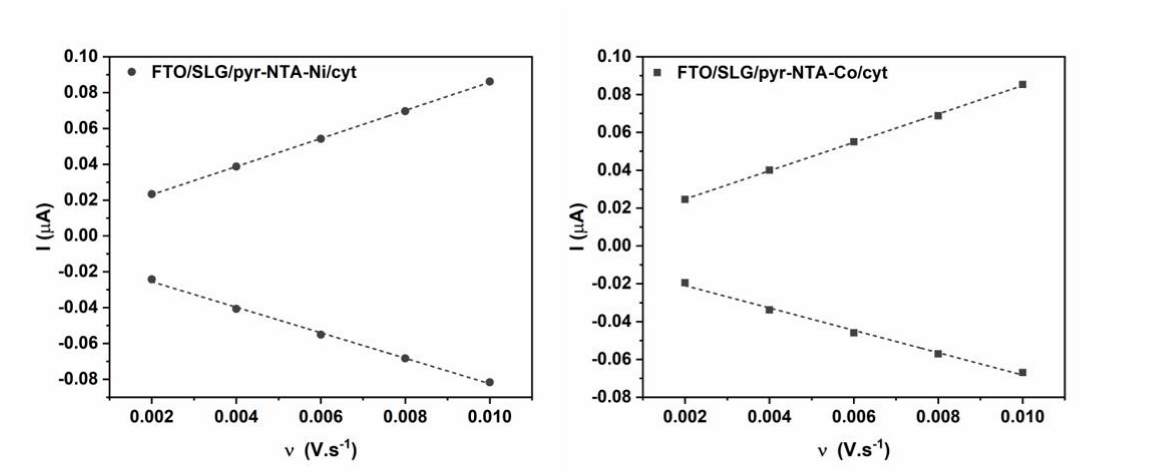

Fig. S3. Linear dependency between scan rate and current intensity of redox peaks for FTO/SLG/pyr-NTA-M^{2+}/cyt nanoassemblies. Ni-based electrode (left) and Co-based electrode (right).

Table. S1. Additional curve fitting results based on R1((R2W1)CPE1) model.

**Sample**	**Illumination**	**Q (10^{-6})**	**Φ**	**W1-R (Ω)**	**W1-T (s)**	**W1-P**	**X^2 (10^{-5})**	**Sum of square (10^{-3})**
**Pyr-NTA-Ni/cyt**	OFF	4.93	0.97	2.62	21.35	0.62	3.47	1.62
**Pyr-NTA-Ni/cyt**	ON	4.92	0.97	2.54	20.81	0.64	6.44	3.02
**Pyr-NTA-Co/cyt**	OFF	4.94	0.97	3.06	21.94	0.60	2.50	1.17
**Pyr-NTA-Co/cyt**	ON	4.96	0.97	3.07	24.32	0.63	5.50	2.58

Computational details

The SLG/SAM/cyt interface has been built with the Maestro2020 suite of programs (Schrödinger Release 2020-4: Maestro, Schrödinger, LLC, New York, NY, 2020). The cytochrome was connected to one NTA molecule *via* coordinate bonds between the Ni^{2+} cation and two nitrogen atoms (labelled cyt-NTA), one from His2 and one from His3 residues, of the His-tag (with His1 connected to the 19AA linker and His6 being the terminal amino acid of the protein). A 6 x 6 x 6 nm^3 simulation box has been build and the cyt-NTA fragment was physisorbed at the SLG centre. Additional 29 NTA-Ni molecules were randomly physisorbed to assure full coverage of the SLG surface. Only one NTA molecule was directly connected to the cytochrome molecule via the two histidine residues, while for the other 29 molecules the coordination of the Ni^{2+} ions has been completed with water molecules of the solvent. The SLG/SAM/cyt interface was embedded in water, described with the TIP3P model, and the charge of the entire system was set as zero by adding Na^+ ions.

The force field parameters for cyt c_{553} have been modified accordingly to account for the haem-His, haem-Cys and haem-Met interaction [1]. For all the systems we considered the central iron atom as Fe(II). SLG parameters were obtained using the procedure reported in our previous work [2]. Parameters for the NTA-Ni-His interactions have been modified accordingly as reported previously [3]. Bonds parameters have been taken from optimised geometry at the DFT/PBE/ LAV2P** level of theory, while charges for the NTA-Ni-His molecule have been obtained at the ESP level of theory. The force field parameters used for the haem and the NTA-Ni-His moieties are available from the authors.

The cubic periodic boundary box was oriented in such a way that the x- and y-axes were taken in the plane of the graphene monolayer, whereas the z-axis was perpendicular to its surface. Periodic boundary conditions were considered in all three dimensions. Electrostatic interactions were treated using the Particle-Mesh Ewald method and bonds were constrained by the LINCS algorithm. Electrostatics and van der Waals (vdW) short-range interaction cut-offs were set to 1.5 nm. Dispersion correction terms were added to account for the Verlet cut-off vdW scheme. Since the graphene was considered periodic in the xy plane, the NVT ensemble was used, with the Nosé−Hoover thermostat, at 300 K and the time constant of 0.5 ps. The graphene monolayer was kept frozen along the x, y and z directions during the entire simulation. The simulation time step was set to 2 fs and the coordinates of all atoms were saved every 10,000 steps, for a total simulation time of 300 ns. The MD simulation was equilibrated after 200 ns, and all the analysis reported here and in the main manuscript refer only to the 200-300 ns time window.

Molecular Dynamics simulations

The interaction of the cyt *c* with the SAM was assessed by means of energy analysis decomposition, in which the non-bonding interactions (van der Waals and Coulomb) were considered. From this analysis we observe that the strongest contribution to the stability of the SLG/SAM interface arises from the vdW interactions, while for the SAM/cyt *c* interface the Coulomb term is predominant (Fig. S4).

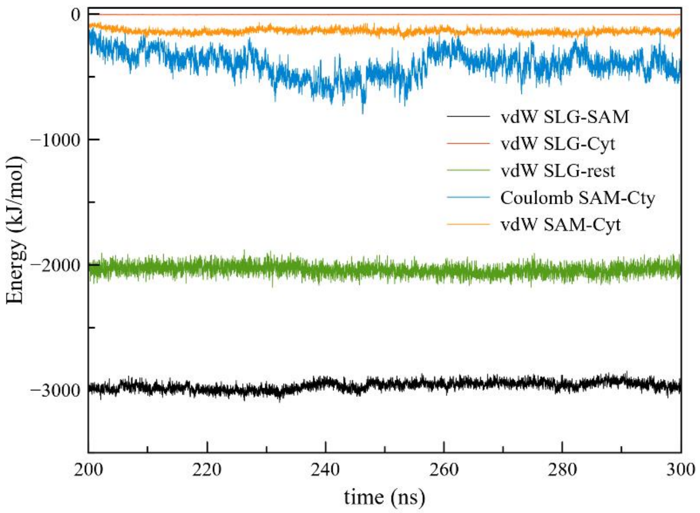

Fig. S4. Energy analysis of the SLG/SAM/cyt interface from the MD simulations. For the sake of clarity, only non-bonding interactions are shown.

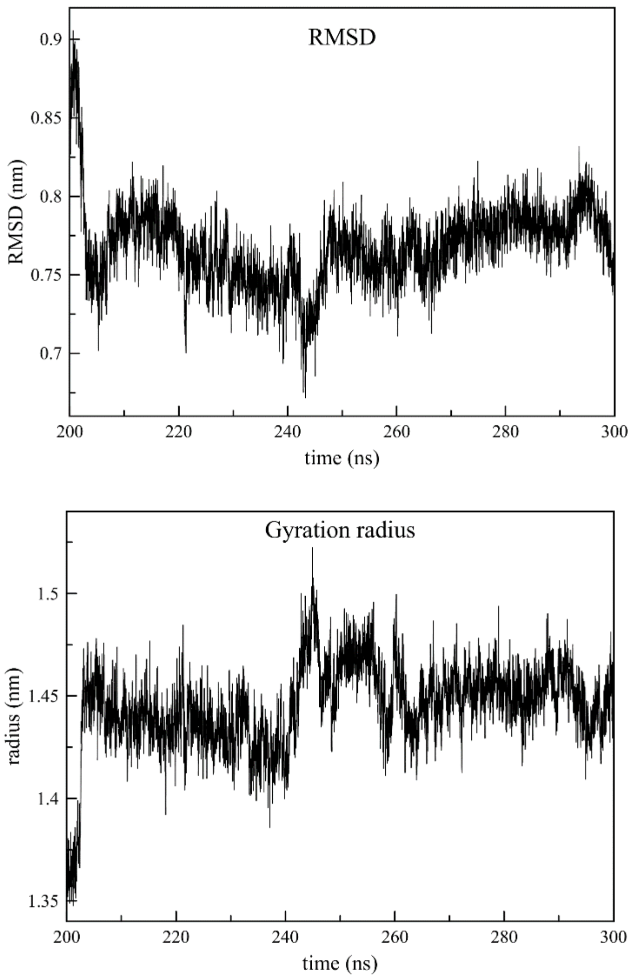

Fig. S5. RMSD and gyration radius of the cyt *c* during the 300 ns MD simulation.

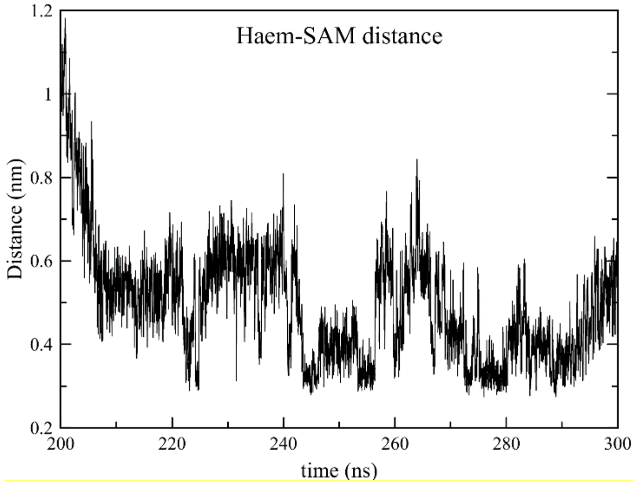

Fig. S6. Minimum distance of the haem/pyr-NTA redox pairs.

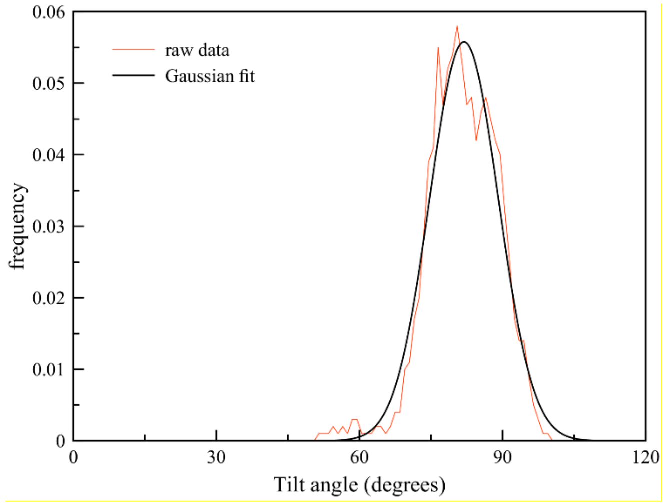

Fig. S7. Distribution of the tilt angle of the haem group with respect to SLG. The tilt angle has been computed as the angle between the haem plane and the SLG surface.

QM/MM calculations

From the last 10 ns of simulation time of the MD trajectory, the minimum distance between the haem and NTA molecules was calculated, and 56 haem/NTA pairs coordinates were extracted. In order to avoid artefacts in the QM calculations, the haem group was neutralised by adding hydrogen atoms to the structure. In our QM/MM scheme the haem/NTA pairs were considered in the QM part, while the entire protein was considered in the MM region as point charges, to account for the anisotropy and effects of the environment on the electronic properties of the QM part. Density functional theory (DFT) ground state single point calculations were performed with the CAM-B3LYP/LACV3P** functional/basis set. The choice of the basis set was dictated by the presence of transition metal elements. The metal centres were considered as Fe(II) and Ni(II) and one carboxylic group of NTA was protonated, to preserve the neutrality of the interface.

From the MD trajectory, the minimum distance between haem and all the SAM molecules was computed, and the closest to haem molecule was selected and their coordinates extracted. In total, 56 frames have been extracted, with an average haem-SAM distance of 0.5 nm, consistent with the MD analysis. Of them, 48 came from an NTA molecule (*major set*), while 8 structures came from a different, neighbouring NTA (*minor set*). Although the minor set consist of only few frames, it is important to consider it in here, as it shows the dynamic behaviour of the cyt protein over the SAM. We stress here that the NTA molecules considered in the analysis are not connected to the His-tag. The QM/MM calculations were performed for both Ni^{2+} and Co^{2+} cations. As the SAM is only marginally affected by the presence of a different metal centre for the MD analysis, the same frames extracted when Ni is present has been considered, substituting the metal centre to Co.

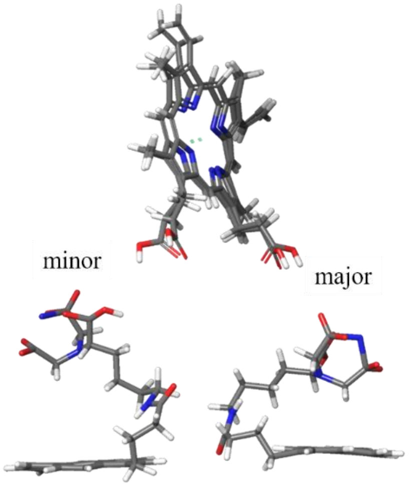

Fig. S8. Representative orientations of the extracted pyr-NTA/haem redox pairs considering the two electronic distributions.

Minor set analysis

As seen in Fig. S9**,** for the minor set the HOMO levels peak at -3.74 ± 0.23 eV, while the LUMO at -0.63 ± 0.40 eV. This results in a relatively large energy gap of 3.11 ± 0.38 eV, much higher than for the major set, of 0.52 eV. This, in turn, will affect the device performance. This can be rationalised by the different localisation of the HOMO and LUMO orbitals over different fragments of the interface, as the HOMO is localised over the porphyrin moiety of the haem group and the LUMO on the NTA-Ni moiety of the SAM molecule. The DET for this set is from haem to pyr-NTA-Ni, as already observed for the major set.

When Co is considered as the coordinating metal centre, a radical system is obtained. For the minor set, HOMO and SOMO orbitals are quasi-degenerated and with similar energy values of -3.78 and -3.76 eV for the HOMO and SOMO, respectively, similar to the major set. On the other hand, the energy of LUMO is -0.43 eV, leading to the energy gap of 3.43 eV.

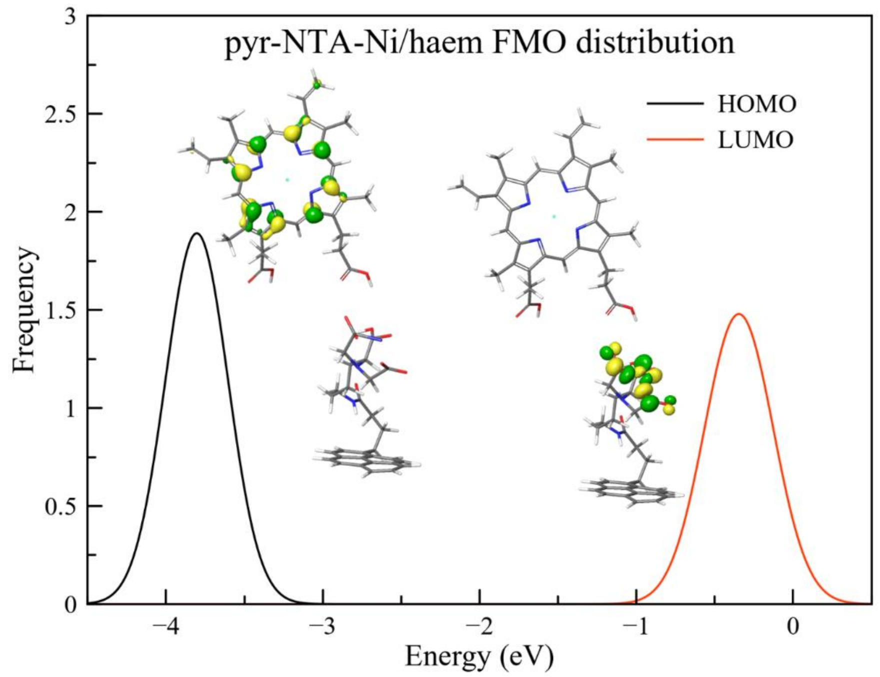

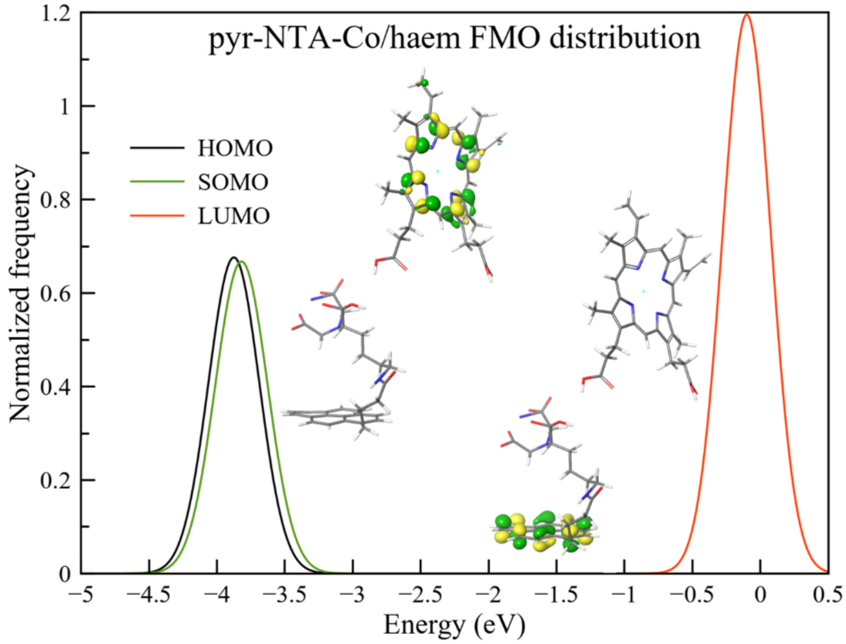

Fig. S9. Frontier molecular orbitals for the pyr-NTA-Ni/haem (top) and pyr-NTA-Co/haem (bottom) interfaces and their electronic distribution.

Dissection of the DET mechanism

The rate of ET steps (k_{et}) is described by the semiclassical Marcus theory for nonadiabatic electron transfer [4]:

$$k_{et} = \frac{2\pi}{\hbar} V^2 \frac{1}{\sqrt{4\pi\lambda kT}} \exp\left(\frac{-(\Delta G + \lambda)^2}{4\lambda kT}\right)$$

The key parameters controlling the ET rate are the electronic coupling V, the reorganisation energy λ and the driving force ΔG. The electronic coupling exponentially decays with the D-A distance (R) as [5]:

$$V \sim V^0 \exp\left[-\frac{\beta}{2}(R - R_0)\right]$$

β is determined by the D-A superexchange interaction, together with their surroundings [6]. Generally, for proteins β is found in the 1.0-1.4 $Å^{-1}$ range, and the thermal average of the different accessible protein configurations strongly decreases the D-A distance effect on the coupling. The reorganisation energy is often treated as an adjustable parameter for proteins, in the 0.5-1.5 eV range [7]. ΔG is defined as the difference between the donor (D) and acceptor (A) redox potential obtained from CV measurements, and for proteins it depends on the environment (water molecules, ions) in the vicinity of the redox centre [8,9].

In a schematic way (Fig. S10), the ET process can be considered either as direct electron tunnelling D →A or two-step hopping D →X → A where thermal D →X activation is followed by the exergonic reaction X → A [10].

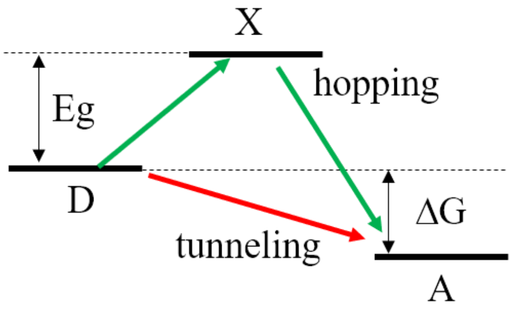

Fig. S10. Schematic representation of the DET tunnelling and hopping mechanisms. ΔG is the driving force; Eg is the energy gap.

References

1 K. Kaszuba, P. A. Postila, O. Cramariuc, M. Sarewicz, A. Osyczka, I. Vattulainen and T. Róg, *Theoretical Chemistry Accounts*, 2013, **132**, 1370.
2 A. M. Kowalska, B. Trzaskowski and S. Osella, *J. Phys. Chem. C*, 2018, **122**, 29405–29413.
3 D. Adam, *ChemSusChem*.
4 R. A. Marcus and N. Sutin, *Biochimica et Biophysica Acta (BBA) - Reviews on Bioenergetics*, 1985, **811**, 265–322.
5 J. C. Genereux and J. K. Barton, *Chem. Rev.*, 2010, **110**, 1642–1662.
6 D. Beratan, J. Betts and J. Onuchic, *Science*, 1991, **252**, 1285.
7 C. C. Moser, T. A. Farid, S. E. Chobot and P. L. Dutton, *Biochim Biophys Acta*, 2006, **1757**, 1096–1109.
8 M. Cascella, A. Magistrato, I. Tavernelli, P. Carloni and U. Rothlisberger, *Proc Natl Acad Sci USA*, 2006, **103**, 19641.
9 A. Warshel, P. K. Sharma, M. Kato, Y. Xiang, H. Liu and M. H. M. Olsson, *Chem. Rev.*, 2006, **106**, 3210–3235.
10 A. A. Voityuk, *J. Phys. Chem. B*, 2011, **115**, 12202–12207.
